# Longitudinal Study on Low-Dose Aspirin versus Placebo Administration in Silent Brain Infarcts: The Silence Study

**DOI:** 10.1155/2018/7532403

**Published:** 2018-10-03

**Authors:** Ilaria Maestrini, Marta Altieri, Laura Di Clemente, Edoardo Vicenzini, Patrizia Pantano, Eytan Raz, Mauro Silvestrini, Leandro Provinciali, Isabella Paolino, Carmine Marini, Matteo Di Giuseppe, Tommasina Russo, Francesco Federico, Cristiana Coppola, Maria Pia Prontera, Domenico Maria Mezzapesa, Vincenzo Lucivero, Lucilla Parnetti, Paola Sarchielli, Maria Peducci, Domenico Inzitari, Giovanna Carlucci, Carlo Serrati, Carla Zat, Anna Cavallini, Alessandra Persico, Giuseppe Micieli, Stefano Bastianello, Vittorio Di Piero

**Affiliations:** ^1^Department of Human Neuroscience, “Sapienza” University of Rome, Rome, Italy; ^2^IRCSS Neuromed, Pozzilli (IS), Italy; ^3^Neurological Clinic, Department of Experimental and Clinical Medicine, Marche Polytechnic University, Ancona, Italy; ^4^Department of Internal Medicine and Public Health, University of L'Aquila, L'Aquila, Italy; ^5^Department of Neurology and Psychiatrics, University of Bari, Bari, Italy; ^6^Neurologic Clinic, Department of Medical and Surgical Specialties and Public Health, University of Perugia, Perugia, Italy; ^7^Department of NEUROFARBA, University of Florence, Florence, Italy; ^8^Department of Neurology, IRCCS San Martino Hospital-IST, Genoa, Italy; ^9^Neurological Division, Ospedale Civile di Imperia, Imperia, Italy; ^10^Department of Cerebrovascular Diseases, Fondazione “Istituto Neurologico C. Mondino”, Pavia, Italy; ^11^Department of Brain and Behavioural Sciences, University of Pavia, Pavia, Italy

## Abstract

**Background:**

We investigated low-dose aspirin (ASA) efficacy and safety in subjects with silent brain infarcts (SBIs) in preventing new cerebrovascular (CVD) events as well as cognitive impairment.

**Methods:**

We included subjects aged ≥45 years, with at least one SBI and no previous CVD. Subjects were followed up to 4 years assessing CVD and SBI incidence as primary endpoint and as secondary endpoints: (a) cardiovascular and adverse events and (b) cognitive impairment.

**Results:**

Thirty-six subjects received ASA while 47 were untreated. Primary endpoint occurred in 9 controls (19.1%) versus 2 (5.6%) in the ASA group (p=0.10). Secondary endpoints did not differ in the two groups. Only baseline leukoaraiosis predicts primary [OR 5.4 (95%CI 1.3-22.9, p=0.022)] and secondary endpoint-a [3.2 (95%CI 1.1-9.6, p=0.040)] occurrence.

**Conclusions:**

These data show an increase of new CVD events in the untreated group. Despite the study limitations, SBI seems to be a negative prognostic factor and ASA preventive treatment might improve SBI prognosis.** EU Clinical trial **is registered with EudraCT Number: 2005-000996-16; Sponsor Protocol Number: 694/30.06.04.

## 1. Introduction

The importance of early recognition of silent brain infarcts (SBIs), defined as cerebral ischemic lesions without overt clinical presentation [1], has been progressively recognized in the last years. SBIs increase the risk of stroke up to four times [2–6] in general population. The presence of SBIs increased the risk of stroke recurrence also in patients with symptomatic ischemic brain infarction, compared to stroke patients without SBIs [6]. Furthermore, the presence of SBIs more than doubles the risk of dementia, including Alzheimer's disease [3], and is associated with a higher conversion from mild impairment to dementia [3, 5].

Accordingly, it seems appropriate to apply secondary stroke prevention strategies, instead of those of primary prevention, in healthy individuals found to have SBIs [7]. The revised guidelines from the American Heart Association/American Stroke Association have begun to consider SBIs as “an entry point for secondary stroke prevention and an event to be prevented” [8]. Among the modifiable risk factors, a careful control of arterial hypertension, particularly nocturnal pattern alteration and morning surge, is important for SBIs prevention [9, 10]. Evidence from the Northern Manhattan study shows that also increased levels of physical activity are associated with a lower risk of SBIs [11]. Nowadays there is no solid evidence to consider the presence of SBIs per se as an indication for antiplatelet therapy in healthy people with SBIs due to the lack of direct trials [7]. Thus, data on long-term treatment with aspirin (ASA) in healthy people with SBIs for the prevention of new cardiovascular events are urgently needed.

The aim of the present study was to assess, in a population of healthy subjects with SBIs: (i) as a primary endpoint, the effect of ASA on the incidence of SBIs, stroke, and transient ischemic attack (TIA); (ii) as secondary endpoints: (a) the efficacy and tolerability of ASA in the prevention of cardiovascular events as a combined endpoint of total mortality, cardiovascular mortality, nonfatal myocardial infarction (MI), nonfatal stroke, TIA, and SBIs; (b) the incidence of cognitive impairment and the effect of ASA therapy on possible development of cognitive impairment.

## 2. Materials and Methods

This longitudinal, randomised, double blind controlled versus placebo study was conducted in eight Italian centres. All consecutive subjects attending the neurological clinic, aged ≥45 years old, who presented at least one SBI at Magnetic Resonance Imaging (MRI) were enrolled.

Exclusion criteria were as follows: (i) presence of stroke or TIA; (ii) contraindication to ASA use; (iii) presence of microbleeds; (iv) indication to anticoagulant therapy; (v) malignant arterial hypertension; (vi) cardiac heart failure (IV class NYHA), (vii) haemoglobin value ≤8g/dl; (viii) platelet count <100.000; (ix) haemorrhagic disorders; (x) ongoing antiplatelet or anticoagulant therapy; (xi) serious inter-current illness.

At baseline, subjects underwent a complete and standardized vascular screening as well as a neuropsychological assessment. Patients were randomized to receive one of two treatments: (a) ASA 100 mg, administered as an enteric-coated white tablet, or (b) placebo, an enteric-coated white tablet with identical appearance. Treatment allocation remained blinded to investigators and subjects until the conclusion of the study, except for patients who withdraw for any collateral or adverse effect and/or any new cardiovascular events.

Treatment to control vascular risk factors was administered to all eligible patients at the screening visit and throughout the study, in accordance with international guidelines [8].

A group of patients were not enrolled in the study because they refused to participate in the pharmacological trial. These subjects were studied prospectively and underwent the same baseline screening and flow chart of exams of those who were randomized.

After the inclusion in the study, all patients were followed for four years and also the ones who dropped-out for any reason except for consensus withdrawal. The annual follow-up included the following: standardized MRI, neuropsychological assessment, blood test, and clinical evaluation. At baseline, at 24 and 48 months, an extracranial carotid duplex, transcranial Doppler, and transcranial colour duplex were performed.

### 2.1. MRI Protocol for Diagnosis of SBIs

At admission, all participants underwent a brain 1.5-Tesla MRI with a standardised protocol for all centres, as previously described [12]. They were positioned comfortably so as to avoid even minimal movements of the head. A scout in the three spatial plans was performed, positioning the sagittal scans on the median line, providing a better visualization of the corpus callosum. Scans have been positioned on axial plane, parallel to the lower margin of the corpus callosum, exploring the whole brain. The following sequences were performed (1-2 acquisitions for each sequence): diffusion-weighted imaging (DWI); TSE double-echo T2 weighted (Proton density-T2) (TR 2000-4500, TE 15-50/80-120); f-FLAIR (TR 7000-11000, TE 150/200, TI 1500-2000, Echo-train-length 30-50); gradient-echo (FFE-FLASH) (TR 600-800, TE 20-30, Flip angle 15-25); and three-dimesional-T1 (SPGR or MPRAGE) (TR 20-30, TE 5-10, Flip angle 50). For all scans, the same number of slices were obtained using the following parameters: 44-48 slices of the thickness of 3 mm (gap: 0 mm), FOV 25 cm, matrix 256 x 256, and L/R direction of coding phase.

SBIs were defined as focal hyperintensity on T2-weighted images, 3 mm in size or larger as described by Vermeer and collaborators [2]. Proton-density sequence was used to distinguish infarcts from dilated perivascular spaces. Infarct lesions in the white matter were distinguished from white matter lesions by corresponding hypointensity on T1-weighted images.

A neuroradiologist, blinded to the patient's medical history, classified SBIs according to size and location. Cortical and subcortical atrophy and leukoaraiosis were evaluated and scored as absent or present. A good “inter-intra-rate reliability” (k=0.70) among three expert neuroradiologists was reached before starting the study enrolment (P.P., F.F., and S.B.).

An experienced neurologist, blinded to MRI results, reviewed the medical history to exclude any previous cerebrovascular overt episode. Finally, medical history and imaging data were matched in order to categorize the infarct as silent or symptomatic.

### 2.2. Evaluation of Cognitive Performance

The neuropsychological evaluation was assessed by de Groot and colleagues' method [13] due to its sensitivity to subcortical dysfunction [14]. Three domains were explored: speed of cognitive processes, memory function, and global cognitive function [14].

To evaluate speed of mental processes, we used the Stroop test, the Paper-and-Pencil Memory Scanning Task, the Letter-Digit Substitution Task, and a verbal fluency test. Memory function was evaluated by a 15-word verbal learning test. As measures of global cognitive function, we used a combination of the above-mentioned tests as well as the Mini-mental State Examination (MMSE).

### 2.3. Clinical Assessment

We recorded (i) demographic data (sex, age, Body Mass Index [BMI], and education level); (ii) vascular risk factors and related treatments (arterial hypertension, diabetes mellitus, hypercholesterolemia, hypertriglyceridemia, current and previous smoking habit, alcohol consumption, hyperhomocysteinemia, internal carotid artery stenosis >50% of the lumen, and intima-media thickness); (iii) medical history (MI, atrial fibrillation [AF], heart failure, metabolic syndrome, ongoing oral anticoagulant or antiplatelet therapy, and migraine with and without aura); (iv) baseline vital signs (systolic and diastolic blood pressure and temperature).

### 2.4. Endpoints

The primary criterion was a combined endpoint of ischemic stroke (IS), TIA, and new SBIs detected at MRI. The evaluation was performed by considering the number of new SBIs occurring during the study, calculated as the difference between lesions at endpoint and baseline MRI. Any TIA or IS occurrence was added to this computation.

The secondary criterion was assessed by (a) the incidence of new cardiovascular events (combined endpoint of total mortality, cardiovascular mortality, nonfatal MI, nonfatal IS, and TIA) and SBIs and the count of adverse events; (b) the eventual cognitive decline. Adverse events were classified among serious or not serious, expected or unexpected, and categorized. The incidence of haemorrhagic stroke and major bleeding of gastrointestinal tract was calculated separately.

### 2.5. Statistics

In order to improve the statistical power of the study, subjects who participate to pharmacological trial were analysed together with those who participate in the observational study. All subjects were divided into two groups according to their decision of starting or not ASA therapy: ASA versus controls (placebo or no therapy).

Descriptive statistics are reported as count and percentage. Categorical data were evaluated with the Chi-square or the Fisher exact test as appropriate. Two predictive models were carried out to assess any potential prognostic factor of new SBIs, stroke, TIA (primary endpoint), or all cardiovascular and adverse events (secondary endpoints). The predictive models have been assessed using the multivariable logistic regression with a forward approach. Results are presented as Odds Ratios (OR) and 95% Confidence Intervals (CI). The predictive models' goodness of fit was tested with the Hosmer-Lemeshow test. Area under the Curve (AUC) values were assessed for each significant variable and the full models. Models sensitivity, specificity, and predictive values were given. Psychomotor speed, memory performance, and cognitive indexes were obtained according to De Groot and colleagues [13]. These scores were compared both transversally and longitudinally using the Wilcoxon Mann-Whitney test and the repeated-measures ANOVA (ANOVARM). ANOVARM models accounted for both between and within sources of variability, using mixed effect models. Effects tested were treatment (2 levels: ASA and controls), visit (5 levels: 0, 12, 24, 36, and 48 months), and the interaction between treatment and visit. All analyses were carried out on an Intent-to-Treat (ITT) population. Analyses pertaining the cognitive evaluation were applied on (1) observed data (no imputation) and (2) last observation carried forward (LOCF) datasets. The ANOVARM analyses conducted on the observed data dataset were adjusted for missing evaluations using the pattern mixture model. All tests were two-tailed with significance set to alpha=0.05 and CI =95%. Data were analysed using the Statistical Analysis System (SAS Institute, Inc., Cary, NC, U.S.A.) package for PC (version 9.2) and Statistical Package for the Social Sciences (SPSS) package for Windows (version 20.0).

Given the low statistical power of the study, a parallel Bayesian analysis was performed on the primary outcome and MI occurrence (composite vascular endpoint) at 1 year for the 50 subjects who were included in the pharmacological trial. We used the Gibbs sampling (a Markov Chain Monte Carlo MCMC algorithm) for obtaining a sequence of random samples (1000) from the multivariate probability distribution and from the joint probability distribution of the uninformative prior and the binomial distribution (successes and failures occurrence) of the data observed.

### 2.6. Ethics

The study was approved by the local Ethics Committee (EU Clinical trial registration: EudraCT Number: 2005-000996-16; Sponsor Protocol Number: 694/30.06.04). The procedures described in the study according to conduction, evaluation, and documentation were conceived in conformity to Good Clinical Practice Guidelines and were inspired by the principles of the Declaration of Helsinki (1964) and its later amendments. All participants to the study signed a written informed consent.

## 3. Results

### 3.1. General Features

During the study period, 350 subjects underwent the baseline screening procedure. At the end, 124 subjects were recruited. Forty-one subjects were excluded after central neuroradiological diagnosis review. Fifty subjects were enrolled in the double-blind study (14 in Perugia, 12 in Roma, 7 in Pavia, 6 in Ancona, 6 in Bari, 1 in Firenze, 1 in Imperia, and 3 in L'Aquila). Out of 50, 24 subjects were randomized to ASA treatment and 26 to placebo. Thirty-three subjects (24 in Roma, 4 in Ancona, 4 in L'Aquila, and 1 in Perugia) were studied prospectively but they did not enter the study because refused to participate in the pharmacological trial. Out of 33, 12 subjects underwent treatment with ASA and 21 with no ASA.

### 3.2. Main Characteristics of the Study Population

The demographical and clinical characteristics of the subjects underwent treatment with ASA and controls are reported in Table 1. Only treatment with Angiotensin-Converting-Enzyme inhibitors resulted in being unbalanced in the two groups (p=0.001). Although significance was not reached, a slight positive trend was observed in control group than in ASA group for greater occurrence of arterial hypertension (29 [61.7%] versus 17 [47.2%] patients, p=0.077, respectively) as well as hyperhomocysteinemia (12 [25.5%] versus 6 [16.7%] patients, p=0.188, respectively).

### 3.3. Primary Endpoint

The number and rates of primary endpoint occurrences were reported in Table 2. Although significance was not reached (p=0.103), there were 9 (19.1%) versus 2 (5.6%) cerebrovascular events and new SBIs events in the control and ASA arms, respectively.

### 3.4. Secondary Endpoint-a

All cardiovascular events occurred in the ASA and control groups during the four years of observation are reported in Table 3. The only, nonsignificant, imbalance was on the primary endpoint, in fact other cardiovascular events (nonfatal MI, all cardiovascular mortality) are fairly balanced 5.6% and 4.2%, respectively, in the ASA and the control groups. Also adverse events are fairly balanced in two groups 5.6% and 4.2%, respectively, in the ASA and the control groups (see Table 3).

All events that led to discontinuation of the treatment are resumed in Table 4. During the study period, two control participants started aspirin and two participants in the ASA group stopped aspirin because of gastrointestinal adverse events.

### 3.5. Logistic Regression Analyses

All demographical and clinical variables reported in Table 1 were then correlated with the primary (model A) and secondary (model B) endpoints with two multivariable logistic models using the forward approach. The only variable retained significance in both model A (primary endpoint) and model B (secondary endpoint) was leukoaraiosis, with OR 5.4 (95%CI 1.3-22.9), p=0.022 and OR 3.2 (95%CI 1.1-9.6), p=0.040, respectively.

Model A was almost discretely predictive (AUC=0.697) with a sensitivity of 72.7%, specificity of 66.7%; positive and negative predictive value of 25.0% and 92.3%, respectively. Conversely, model B was modestly predictive (AUC=0.644), with a sensitivity of 61.1%, specificity of 67.7%, and positive and negative predictive value of 34.4% and 86.3% respectively.

### 3.6. Secondary Endpoint-b

Changes (Δ) in psychomotor speed, memory performance, and global cognitive indexes between the last and the first visit after assignment to the drug for both ITT and completer populations were reported in Table 5. No significant differences between groups were detected.

ANOVARM model's results are reported in Table 6. The effect of “treatment” and the interaction between “treatment” and “visit” were nonsignificant in each model performed.

### 3.7. Bayesian Analysis on the Composite Vascular Endpoint

An exploratory Bayesian analysis was performed excluding the nonrandomized patients to increase the power of the analysis. The data at the end of the first year only were considered in this analysis because almost all patients completed follow-up at this time point. According to 1000 simulations there was a 96.3% chance of achieving higher failure rates on the composite vascular endpoint (primary endpoint + MI occurrence) with placebo with a 95% chance the difference in the proportion of failure between placebo and ASA falling in the range (+38.0%, -1.4%) and a median value of +15.2% (Table 7). In practice for 963 samples of the 1000 simulated, the number of placebo failures was greater than the number of ASA failures (Table 7).

## 4. Discussion

Our study has shown that (i) although significance was not reached, an increase of new CVD events in controls occurred, (ii) there was no difference in tolerability between ASA and control group, (iii) presence of leukoaraiosis at baseline was independently associated with the occurrence of primary and secondary endpoints, and (iv) there was no significant difference in incidence of cognitive impairment between ASA and control group during the follow-up.

The strengths of our study were (i) prospectively multicentre data collection, with a homogeneous and aggressive preventive vascular treatment, (ii) use of strict radiological and clinical inclusion criteria, and (iii) central reading of neuroimaging, so as to avoid diagnostic bias. Indeed, the variation in MRI characteristics and imaging criteria for SBIs diagnosis may partially account for discrepancies in various studies and consequently SBIs detection [5, 15]. A meta-analysis on radiological criteria for SBIs diagnosis has underlined that, in half of studies published, SBIs were defined simply as hypointense area on T1 scans and hyperintense area on T2-weighted images sized ≥3 mm, not considering exclusion criteria for dilated Virchow-Robin spaces, leading to a consequently possible overestimation of SBIs prevalence [15]. Moreover, considering the established association between vascular risk factors and SBIs [1], the homogeneous treatment of all subjects in terms of vascular prevention allowed minimizing their possible role in CVD incidence and better evaluating the role of antithrombotic therapy.

The major limit of our study is the small size of population, mainly due to (i) the enrolment method since subjects spontaneously came to clinical observation for other reasons (mainly headache or nonspecific dizziness), (ii) strict inclusion criteria, and (iii) the consent to randomization. Indeed, randomization was often hindered by general practitioners who preferred to prescribe directly ASA treatment. Although data to guide management of patients with silent infarction are limited, the guideline American Heart Association/American Stroke Association summarizes these data where they could be found and incorporate them into relevant sections of this guidelines [8]. Nevertheless, antithrombotic therapy in subjects with SBIs is still to be supported by clinical trials [7, 16].

Despite the small size of our population, ASA therapy could ameliorate SBIs prognosis on CVD. This finding is in line with other trials investigating the preventive effect of antithrombotic agents. Two small randomised controlled Japanese trials examining SBIs as a surrogate endpoint have been carried out in selected diabetic population, mostly free of SBIs at baseline, treated with either the antithrombotic agents cilostazol [17] or dilazep hydrochloride [18]. Both trials found that the incidence of SBIs was significantly lower in the drug-treated group than in the control group. In patients with nonvalvular AF, aspirin was found to reduce SBIs [19], while anticoagulant therapy did not affect the rate of SBIs in the SPINAF study [20]. If ASA preventive treatment might contribute to ameliorate SBI, prognosis will be better clarified by ongoing trials as ASPREE study [21] and its substudy ENVIS-ion [22] aims to determine if a low dose of aspirin may prevent death and disability, including cognitive decline, and reduce the development of white matter hyperintense lesions and SBIs as assessed by MRI in elderly, as well [22].

Furthermore, we observed a trend towards a greater occurrence of arterial hypertension and hyperhomocysteinemia in controls than in ASA group. Despite the lack of statistically significant differences between the two groups, we cannot exclude that this might slightly bias our results.

Baseline characteristics of our study population confirmed the strong association between SBIs and arterial hypertension [1, 23] and, to lesser extent, hypercholesterolemia [1]. Indeed, more than half and around two-thirds of population at baseline were affected by arterial hypertension and hypercholesterolemia, respectively. On the other hand, only few subjects presented hyperhomocysteinemia [1, 23] and smoking habit [1], also found to be associated with SBIs, and others risk factors associated with symptomatic lacunar infarction as diabetes and ischemic heart disease [23].

The vast majority of SBIs is small and deep and reflects penetrating artery disease, a pathogenesis shared with lacunar infarcts. Ischaemic leukoaraiosis, or white matter hyperintensities, is thought to be, together with lacunar infarction, different form of small vessel disease [24]. The presence and extent of leukoaraiosis represent a radiological marker of small vessel disease and an important predictor of first-ever and recurrent stroke, cognitive impairment, and functional disability [25]. It is recently used as surrogate endpoint in CVD clinical trials [26]. In our study, the presence of leukoaraiosis is associated with primary and, to lesser extent, secondary endpoint. This finding might confirm the belonging of leukoaraiosis and SBIs to the same spectrum of pathology, possibly being different temporal stages of the same pathology [27, 28]. The brain mapping in patients with leukoaraiosis showed that tiny clinically silent acute infarcts occur in these patients [27]. The radiological characteristic of these lesions became similar to characteristics of preexisting leukoaraiosis over time, suggesting that the accumulation of SBIs could be one of the most important, if not the primary cause of leukoaraiosis [27].

In our population, there was no significant difference in the incidence of cognitive impairment between patients treated and not during the 4-year follow-up. The presence of SBIs is known to be associated with a 2-fold risk of dementia, a steeper decline in age-associated cognitive function and a higher conversion from mild impairment to dementia [3, 5]. The question of whether low-dose aspirin might be protective against cognitive decline remains unanswered [29, 30]. Possible explanations of the lack of long-term aspirin treatment effect in our population could be (i) the relative young age (median of 66 years in the ASA group versus 68 years in the control group), (ii) the relative short-term follow-up, and (iii) the strict control during all the follow-up of vascular risk factors, emerged as important contributors to the development of Alzheimer's disease.

The general practitioners' attitude to treat in secondary prevention healthy subjects with SBIs in daily clinical practice has preceded of some years the experts' agreement. In fact, recently American Heart Association/American Stroke Association guidelines reported SBIs as an important and emerging issue in secondary stroke prevention.

Despite the fact that larger randomized studies are needed to confirm these findings in subjects with silent brain infarcts, these results may suggest that subjects with SBI are at risk of cardiovascular events and benefit from secondary prevention therapy. A further observation emerging from this study is that also subjects with mild leukoaraiosis are at risk of cardiovascular events and probably have to be treated in secondary prevention too. These subjects need to be followed up as well as stroke patients.

## Figures and Tables

**Table 1:** **Baseline demographical and clinical characteristics of the study population (n=83) and bivariate comparison between patients treated and not treated**. ^*∗*^Value given as median (interquartile range). All other data are reported as absolute number of subjects (%).

	**ASA**	**Controls**	**p value**
	**n=36**	**n=47**	
**Demographic characteristics**			
Female sex	24 (66.7)	33 (70.2)	0.730
Age*∗*	66 (54-72)	68 (60-73)	0.429
Education ≥9 years	11 (32.4)	18 (42.9)	0.582
Body Mass Index			0.551
25-30	18 (50.0)	13 (27.6)	
≥30	6 (16.7)	9 (19.1)	
**Risk factors**			
Arterial hypertension	17 (47.2)	29 (61.7)	0.188
Diabetes mellitus	1 (2.8)	1 (2.1)	1.000
Hypercholesterolemia	26 (72.2)	29 (61.7)	0.315
Hypertriglyceridemia	8 (22.2)	5 (10.6)	0.150
Current smoking	6 (16.7)	7 (14.9)	0.826
Previous smoking habit	11 (30.6)	10 (21.3)	0.335
Excessive Alcohol consumption	9 (25.0)	11 (23.4)	0.866
Hyperhomocysteinemia	6 (16.7)	12 (25.5)	0.077
Carotid atheroma	3 (8.3)	1 (2.1)	0.312
**Medical history**			
History of atrial fibrillation	1 (2.8)	-	0.434
Ischemic Heart Disease	1 (2.8)	-	0.434
Heart failure	-	-	-
Migraine with aura	4 (11.1)	2 (4.3)	0.396
Migraine without aura	7 (19.4)	12 (25.5)	0.513
**Treatments**			
Statins	6 (16.7)	7 (14.9)	0.826
Fibrate	1 (2.8)	-	0.434
ACE inhibitors	13 (36.1)	3 (6.4)	**0.001**
ARB	6 (16.7)	15 (31.9)	0.113
Beta-blockers	6 (16.7)	5 (10.6)	0.422
Calcium channel blockers	3 (8.3)	10 (21.3)	0.135
Diuretics	8 (22.2)	11 (23.4)	0.899
Anti-arrhythmic	-	1 (2.1)	1.000
Oral hypoglycaemic	-	2 (4.3)	0.503
Insulin	-	-	-
Nitrated	2 (5.6)	-	0.185
**Lesion location**			
Basal ganglia	5 (13.9)	11 (23.4)	0.276
Cerebellum	2 (5.6)	2 (4.3)	1.000
Brainstem	3 (8.3)	10 (21.3)	0.135
Sub-cortical	29 (80.6)	39 (83.0)	0.776
Peri-ventricular	23 (63.9)	32 (68.1)	0.689
Leukoaraiosis	12 (33.3)	19 (40.4)	0.508
Cortical atrophy	12 (33.3)	12 (25.5)	0.437
Sub-cortical atrophy	10 (27.8)	6 (12.8)	0.086
Right side	31 (86.1)	43 (91.5)	0.492
Left side	34 (94.4)	4 (89.4)	0.693
**Cognitive and behavioural tests**			
ADL score*∗*	6 (6-6)	6 (6-6)	0.862
IADL score*∗*	8 (8-8)	8 (8-8)	0.134
MMSE score*∗*	29 (27-30)	29 (27-30)	0.874
BDI score*∗*	3,5 (1,75-7)	5 (1-7)	0.917
HDRS score*∗*	11 (14-15,5)	13 (9-14,5)	0.284
MADRS score*∗*	6 (2,5-10,5)	8 (3-12,5)	0.299
IQ-code score*∗*	81 (78,5-85,5)	81,5 (79-87)	0.476

ACE inhibitors: Angiotensin-Converting-Enzyme inhibitors; ARB: Angiotensin-Receptor Blockers; ADL: Activities of Daily Living; IADL: Instrumental Activities of Daily Living; MMSE: Mini-Mental State Examination; BDI: Beck Depression Inventory; HDRS: Hamilton Depression rating Scale; MADRS: Montgomery-Asberg Depression Rating Scale; IQ-code: Informant Questionnaire on Cognitive Decline in the Elderly.

**Table 2:** Primary endpoint occurrences.

	**ASA n=36**	**controls n=47**	
	**n (%)**	**n (%)**	**p-value (Fisher Exact)**
***New SBIs***	1 (2.8)	6 (12.8)	0.103
***Stroke***	1 (2.8)	2 (4.3)	
***TIA***	-	1 (2.1)	
***No CV events***	34 (94.4)	38 (80.9)	

**Table 3:** Secondary endpoint-a occurrences (all cardiovascular events, SBIs and adverse events).

	**ASA n=36**	**controls n=47**	
	**n (%)**	**n (%)**	**p-value (Chi-Square)**
***Non-fatal stroke***	1 (2.8)	1 (2.1)	0.331
***TIA***	-	1 (2.1)	
***New SBIs***	1 (2.8)	6 (12.8)	
***Non-fatal MI***	2 (5.6)	1 (2.1)	
***CV mortality***	-	1 (2.1)	
***Gastrointestinal adverse events***	2 (5.6)	1 (2.1)	
***Epistaxis***	-	1 (2.1)	
***Other causes of mortality***	-	-	
***No events***	30 (83.3)	35 (74.5)	

**Table 4:** Number and rates of secondary endpoints, tolerability and drop out occurrences during the trial.

	**ASA n=36**	**controls n=47**
	**n (%)**	**n (%)**
***Primary endpoint***	2 (5.6)	9 (19.1)
***Other cardiovascular events***	2 (5.6)	1 (2.1)
***Adverse events***	2 (5.6)	2 (4.2)
***Switch ASA***	-	2 (4.2)
***Lost to follow-up***	9 (25.0)	9 (19.1)
***Mortality***	-	1 (2.1)
***Low compliance***	2 (5.6)	-
***Withdraws consent***	4 (11.1)	-

***Completed follow-up***	15 (41.7)	23 (48.9)

**Table 5:** Secondary endpoint-b: change (last follow-up versus baseline) in cognitive indexes.

	**ASA**	**controls**	**p-value**
**Change in the Psychomotor speed score**			
** LOCF Δ (last-first) Mean (SD)**	-0.03 (0.17)	-0.04 (0.54)	0.3205
** NON IMPUTED Δ (last-first) Mean (SD) **	0.07 (0.05)	0.19 (0.99)	0.3082

**Change in the Memory performance score**			
** LOCF Δ (last-first) Mean (SD)**	-0.29 (0.61)	-0.08 (0.74)	0.2238
** NON IMPUTED Δ (last-first) Mean (SD) **	0.14 (0.41)	-0.08 (0.91)	0.3747

**Change in the Global cognitive index score**			
** LOCF Δ (last-first) Mean (SD)**	-0.20 (0.46)	-0.12 (0.51)	0.2507
** NON IMPUTED Δ (last-first) Mean (SD) **	-0.07 (0.34)	-0.03 (0.61)	0.8353

**Table 6:** ANOVARM models on cognitive domains.

		**Treatment**	**Visit**	**Treatment** **∗** **Visit**
		**p-value**	**p-value**	**p-value**
**Psychomotor speed**	Non imputed	0.1776	0.3640	0.0976
	LOCF	0.2523	0.6886	0.6286
**Memory performance**	Non imputed	0.3744	0.0603	0.8072
	LOCF	0.6512	0.0011	0.3446
**Global cognitive index**	Non imputed	0.8449	0.1548	0.9128
	LOCF	0.9332	<0.0001	0.8932

**General cognitive evaluation**	Non imputed	0.6969	0.3913	0.5367
	LOCF	0.9861	0.0201	0.3556
**Episodic memory**	Non imputed	0.9032	0.3466	0.6365
	LOCF	0.4948	0.0967	0.3351
**Short-term memory**	Non imputed	0.2731	0.9264	0.9663
	LOCF	0.4543	0.9630	0.5808
**Executive functions**	Non imputed	0.8123	0.6211	0.5504
	LOCF	0.0952	0.8617	0.4169
**Language**	Non imputed	0.2712	0.9891	0.9413
	LOCF	0.6422	0.7070	0.8942
**Problem solving**	Non imputed	0.7387	0.7072	0.3618
	LOCF	0.8405	0.0224	0.0677
**Dementia staging**	Non imputed	0.5226	0.1699	0.5337
	LOCF	0.6445	0.7873	0.4778
**Daily living activities**	Non imputed	0.4063	0.9233	0.9826
	LOCF	0.1288	0.9979	0.3036
**Major psychiatric disorders**	Non imputed	0.1139	0.3879	0.1515
	LOCF	0.1028	0.0079	0.4263

**Table 7:** Bayesian analysis on the primary outcome + MI.

**Bayesian analysis**									
	**Quantiles**								
	**1.0%**	**2.5%**	**5.0%**	**25.0%**	**50.0%**	**75.0%**	**95.0%**	**97.5%**	**99.0%**
**% of (placebo failures-ASA failures)**	+38.0%	+34.2%	+31. 0%	+21.4%	+15.2%	+9.4%	+1.3%	*-1.4%*	*-4.8%*

Light face values=ASA better than placebo. Italic values=placebo better than ASA.

## Data Availability

Data are not freely available due to patient privacy.
